# Worldwide hotspots and trends in stem cell therapy for kidney disease in the last decade: a bibliometric and visualization analysis from 2015 to 2024

**DOI:** 10.3389/fimmu.2025.1619291

**Published:** 2025-07-21

**Authors:** Shengchun Liao, Xinyue Zhang, Yiting Zhou, Lingchen Wang, Chi Chen, Chaoyang Ye, Yuan Zhou, Chen Wang

**Affiliations:** ^1^ Department of Nephrology, Shuguang Hospital Affiliated to Shanghai University of Traditional Chinese Medicine, Shanghai, China; ^2^ TCM Institute of Kidney Disease, Shanghai University of Traditional Chinese Medicine, Shanghai, China; ^3^ Shanghai Key Laboratory of Traditional Chinese Clinical Medicine, Shanghai University of Traditional Chinese Medicine, Shanghai, China; ^4^ Department of Nephrology, Seventh People’s Hospital, Shanghai University of Traditional Chinese Medicine, Shanghai, China; ^5^ Department of Endocrinology, Shuguang Hospital Affiliated to Shanghai University of Traditional Chinese Medicine, Shanghai, China

**Keywords:** kidney disease, kidney injury, nephropathy, stem cell therapy, bibliometric

## Abstract

**Background:**

Kidney disease represents a significant global health concern. Stem cell therapy has gained attention as a potential solution for chronic kidney disease, acute kidney injury, and end-stage renal disease. This study aims to provide a comprehensive overview of the status of stem cell therapy for kidney disease through a systematic review of the literature.

**Methods:**

The literature included in this study was exclusively sourced from the Web of Science Core Collection. CiteSpace, VOSviewer, R-Bibliometrix, and the Literature Data Governance and Analysis System to evaluate factors such as publication quantity, author contributions, institutional involvement, geographic distribution, and keyword usage.

**Results:**

This study on stem cell therapy for kidney disease included 1,874 articles. A significant number of publications came from China and the United States. The Mayo Clinic had the highest publication output, while *Stem Cell Research & Therapy* was the leading journal in terms of publication volume. Additionally, Lerman LO was the most prolific author in this field. Currently, there is a growing focus on mesenchymal stem cells and acute kidney injury models in this field. Future research is likely to explore topics such as extracellular vesicle-based therapies, various stem cell types, diabetic nephropathy, and membranous nephropathy.

**Conclusion:**

This study applied bibliometric methods to assess the application and development of stem cell therapy for kidney disease over the past decade. It identified key research areas and forecasted future trends. The findings offer valuable insights for guiding future investigations into stem cell therapy for kidney disease.

## 1 Introduction

Kidney disease represents a significant global health concern, impacting millions of individuals and contributing to high mortality rates and healthcare costs (1–3). Although dialysis and kidney transplantation have improved patient outcomes, these treatments are often limited by factors such as organ shortages, immune rejection, and long-term complications (4). Recently, stem cell therapy have gained attention as a potential solution for chronic kidney disease (CKD), acute kidney injury (AKI), and end-stage renal disease (ESRD) (5). These therapies offer the possibility of regenerating damaged kidney tissue, restoring function, and minimizing the need for invasive procedures (6–8). However, despite promising preclinical results and ongoing clinical trials, the widespread implementation of stem cell-based treatments faces significant challenges, including concerns about safety, efficacy, and variability in patient responses (9–11).

Bibliometric analysis has become an essential tool for examining research trends, emerging areas of interest, and developments in studying various diseases. By utilizing bibliometric techniques to assess publication volume, authorship, and citation impact, researchers can gain valuable insights into the progress, challenges, and opportunities within a field. In recent years, bibliometric studies have focused on the growing body of research concerning stem cell therapy for kidney diseases. These studies have identified key trends, such as the increasing focus on mesenchymal stem cells (MSCs), extracellular vesicles (EVs), and their potential to alleviate kidney injury (12, 13). However, a comprehensive bibliometric analysis of stem cell therapy for kidney disease has yet to be conducted.

The central research question of this bibliometric study is: What are the current trends and gaps in research on stem cell therapy for kidney disease, and how can bibliometric analysis guide future research efforts? This study aims to provide a comprehensive overview of the status of stem cell therapy for kidney disease through a systematic review of the literature. By examining a broad range of studies—covering various types of stem cells, gene therapy, and stem cell transplantation—the research seeks to identify major barriers in the field and suggest strategies to address them. It will help prioritize areas with the most significant potential for clinical impact. Additionally, bibliometric analysis can promote collaboration between institutions and researchers, supporting a global effort to tackle the challenges of stem cell-based therapies for kidney diseases (14).

## 2 Materials and methods

### 2.1 Data retrieval and collection

**Figure 1:**
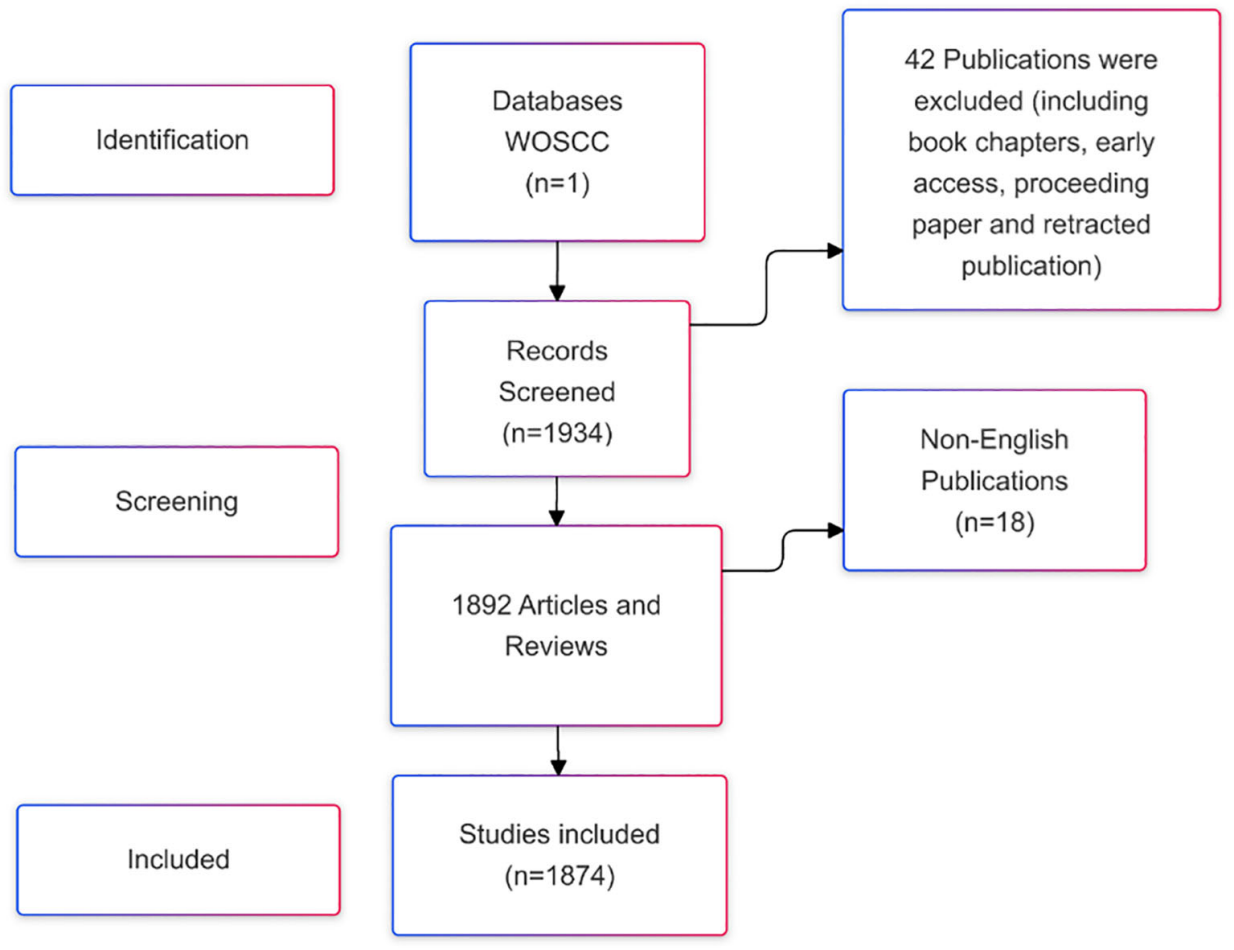
Flowchart of search strategy and screening.

Web of Science is a widely recognized and comprehensive database, hosting over 12,000 reputable academic journals and providing valuable international academic resources (15). The literature included in this study was exclusively sourced from the Web of Science Core Collection (WOSCC) (16). The search strategy employed was: ((TS=(“stem cells” OR “stem cell”) AND TS=(“kidney disease” OR “renal disease” OR “kidney injury” OR ‘nephropathy’ OR ‘nephrosis’) AND TS=(‘therapy’ OR ‘treatment’ OR ‘therapeutics’ OR ‘cure’ OR ‘therapia’)) AND DT=(Article OR Review) AND LA=(English)). The data was retrieved for the period from January 1, 2015, to December 31, 2024. A total of 1,874 publications were initially retrieved, consisting of articles and reviews. The search results were exported as a plain text file and saved in.txt format. The screening and selection process is illustrated in **Figure 1**.

### 2.2 Data analysis

Bibliometrics is a quantitative analysis method that applies mathematical and statistical techniques to examine literature in order to reveal research trends and progress in a specific field. It evaluates factors such as publication quantity, author contributions, institutional involvement, geographic distribution, and keyword usage. In this study, all data retrieved from WOSCC were imported into several tools—CiteSpace (version 6.4.R1), VOSviewer (version 1.6.20), R-Bibliometrix (version 4.3.0), and the Literature Data Governance and Analysis System (Ldgas)—to create visualizations for both quantitative and qualitative analysis. CiteSpace (version 6.4.R1) was used to analyze references and keywords with the strongest citation bursts, perform keyword timeline analysis, create overlay maps of journals, and conduct keyword cluster analysis. VOSviewer (version 1.6.20) was employed to visualize co-occurrence networks for countries, institutions, citations, references, journals, authors, and keywords. Each node in these co-occurrence networks represents a country, institution, publication, author, or keyword, and these nodes are grouped based on their type. R-Bibliometrix (version 4.3.0) was utilized to generate visualizations of general information, including publication volumes by year, country, and journal. Finally, Ldgas was used to analyze the literature further, producing visualizations such as keyword word clouds, publication volume charts for authors and institutions, publication timelines for authors, and collaboration diagrams for countries/regions.

## 3 Results

### 3.1 Basic information and annual publications of the included literature

**Figure 2:**
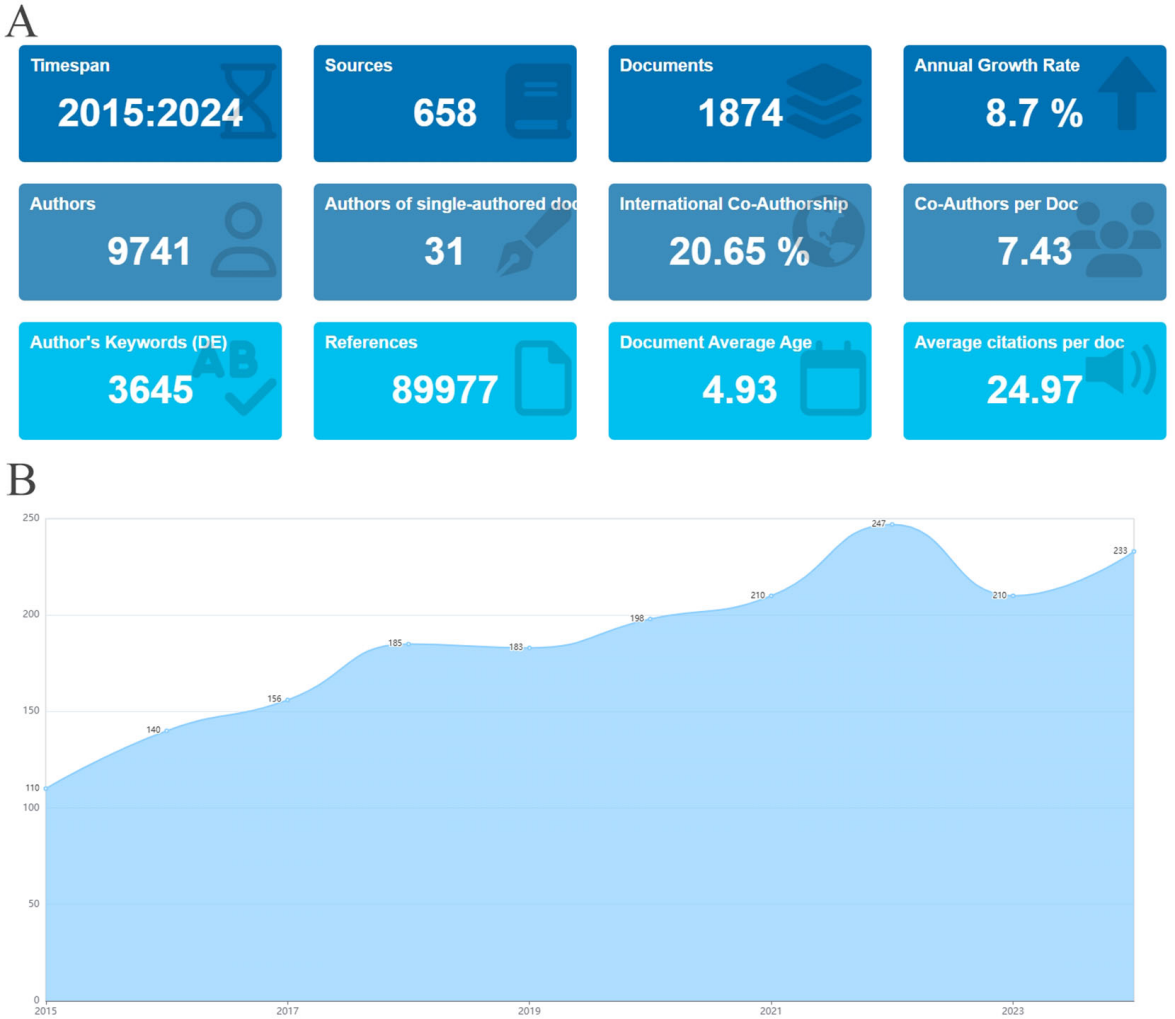
Basic information and annual publications of the included literature. **(A)** Basic information of literature. **(B)** Annual publication volume of literature on stem cell therapy for kidney disease from 2015 to 2024.

An analysis was carried out on the 1,874 articles included in this study on stem cell therapy for kidney disease. The basic information is presented in **Figure 2A**. **Figure 2B** shows the publication trend in this area over the last ten years. Literature production increased steadily from 2015 to 2022, with an exceptionally high output in the past two years. In 2022, the number of publications peaked, highlighting the growing focus on research in this field.

### 3.2 Analysis of geographical distribution and institutions

**Figure 3:**
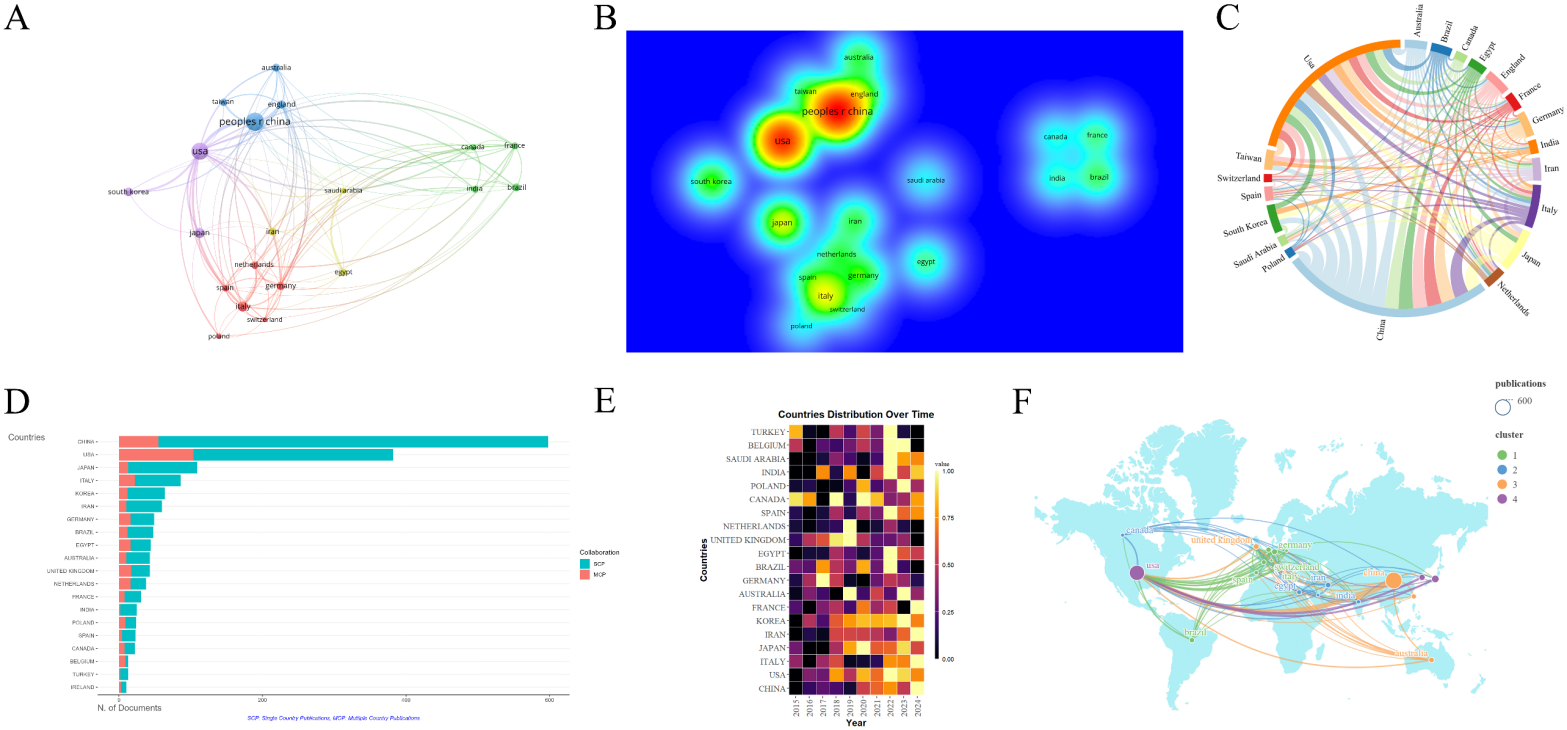
Analysis of countries/regions engaged in research on stem cell therapy for kidney disease. **(A)** Occurrence of contributing countries/regions. **(B)** Density visualization of countries/regions. **(C)** Collaboration chord diagram of countries/regions. **(D)** The top 20 countries’ publication volume chart. **(E)** Annual distribution of publications by countries/regions. **(F)** Countries/regions collaboration map.

**Figure 4:**
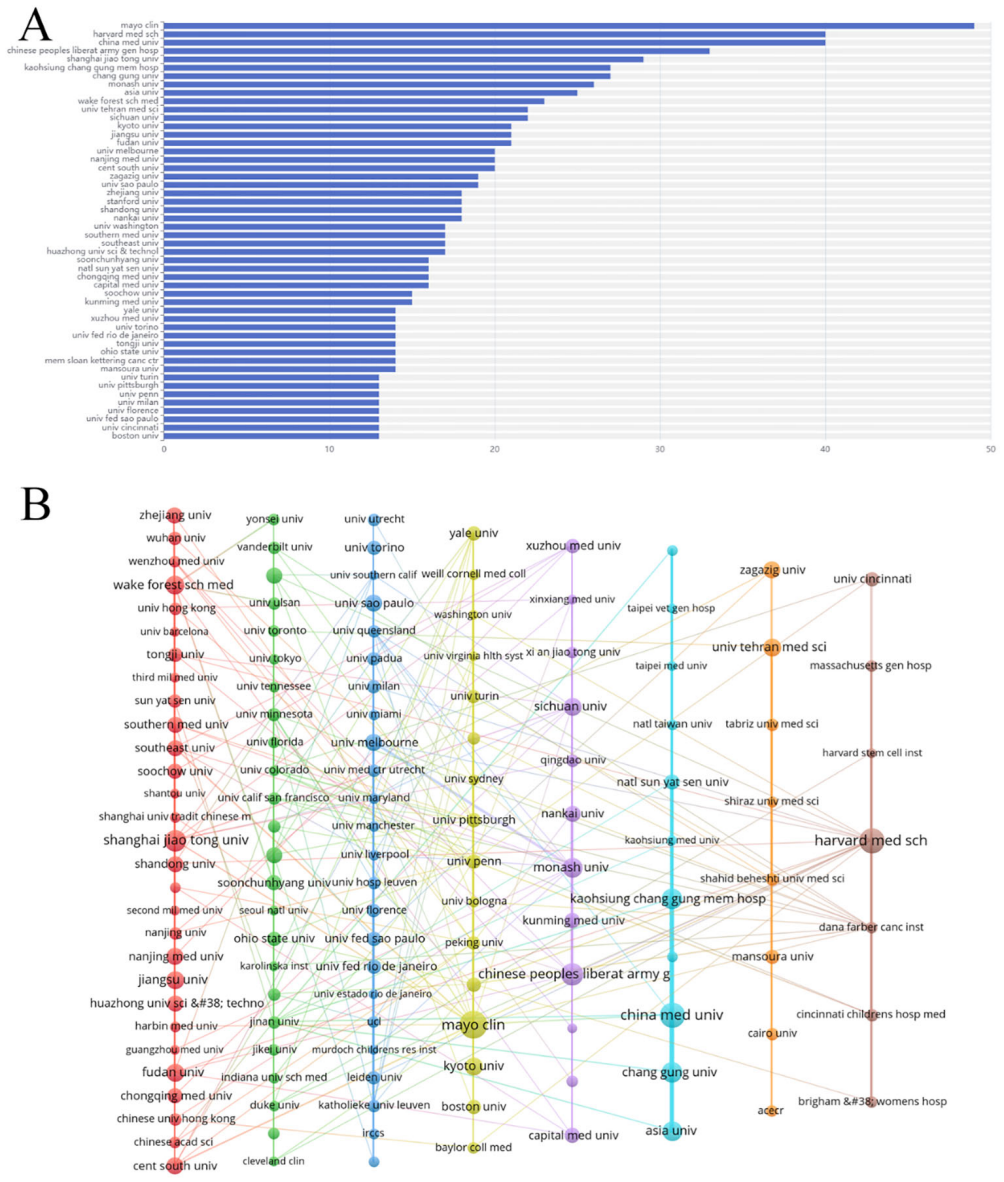
Analysis of institutions engaged in research on stem cell therapy for kidney disease. **(A)** The top 50 institutions’ publication volume chart. **(B)** Network map of institutions.

The literature was distributed across 83 countries and regions. China produced the highest number of publications (592, 31.6%), followed by the United States (497, 26.5%), Italy (121, 6.5%), Japan (120, 6.4%), and South Korea (80, 4.2%). Both China and the United States have a significant number of publications, and research in these regions is particularly prominent (**Figures 3A, B**). The primary research hubs are Asia, Europe, North America, and Australia, with considerable collaboration among these regions. The United States leads in the number of collaborative papers (**Figures 3C, D, F**). Over the past decade, Canada and Turkey were likely pioneers in this field. However, in recent years, research efforts have been more pronounced in other Asian and European regions, as well as in the United States (**Figure 3E**). A total of 335 institutions have contributed to this research. The top five publishing institutions were the Mayo Clinic (49, 2.6%), Harvard Medical School (40, 2.1%), China Medical University (40, 2.1%), Chinese People’s Liberation Army General Hospital (33, 1.8%), and Shanghai Jiaotong University (29, 1.5%). Among the ten institutions with the highest publication numbers, the United States, mainland China, and Taiwan each accounted for three (**Figure 4A**). The collaboration network reveals that China Medical University has established strong connections with institutions in Taiwan, Monash University has deep ties with research centers in China, and several Chinese institutions have collaborated with Harvard Medical School (**Figure 4B**).

### 3.3 Analysis of disciplines and journals

**Figure 5:**
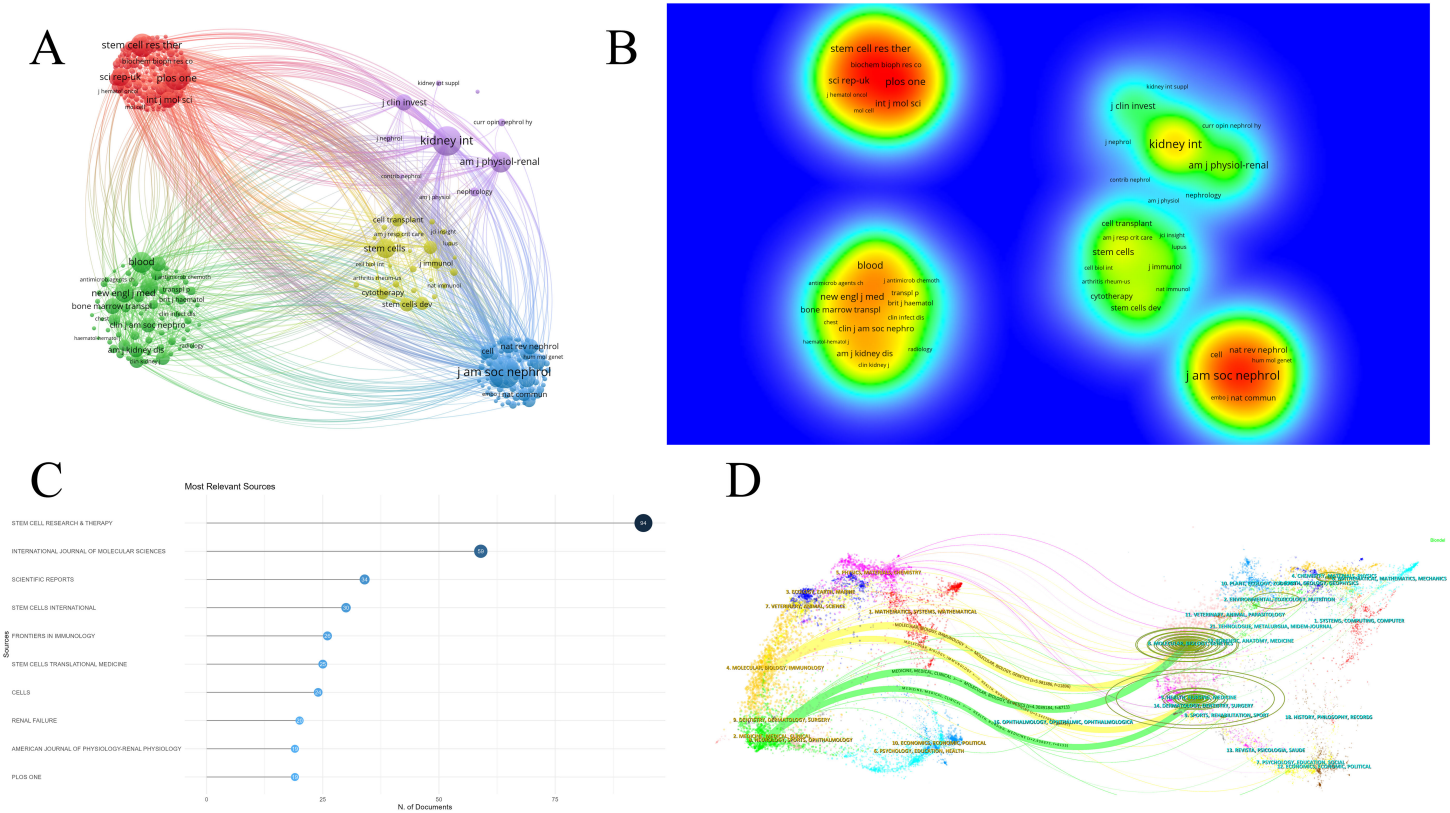
Analysis of journals in research on stem cell therapy for kidney disease. **(A)** Network map of journals. **(B)** Density visualization of journals. **(C)** The top 10 journals’ publication volume chart. **(D)** Overlay map of journals.

The literature analyzed in this study is sourced from 658 journals. **Figure 5A** demonstrates that journals with similar colors tend to have more active citation relationships. **Figure 5B** indicates that journals with warmer color temperatures are associated with higher citation frequencies and more significant academic influence. A total of eight journals have published 20 or more articles in this field. The top five journals by publication volume were *Stem Cell Research & Therapy* (IF 7.1), *International Journal of Molecular Science* (IF 4.9), *Scientific Reports* (IF 3.8), *Stem Cells International* (IF 3.8), and *Frontiers in Immunology* (IF 5.7) (**Figure 5C**). The overlay map of journals illustrates the citation relationships between citing and cited journals, with the left side representing citing journals and the right side representing cited journals (17). As shown in **Figure 5D**, the labels correspond to the disciplines covered by the journals, and the colored paths represent citation relationships. The two yellow paths indicate that research published in journals focused on Molecular/Biology/Genetics and Health/Nursing/Medicine is frequently cited by journals in Molecular/Biology/Immunology. Similarly, the two green paths suggest that research from journals in Molecular/Biology/Genetics and Health/Nursing/Medicine is often cited by journals in Medicine/Medical/Clinical.

### 3.4 Analysis of authors

**Figure 6:**
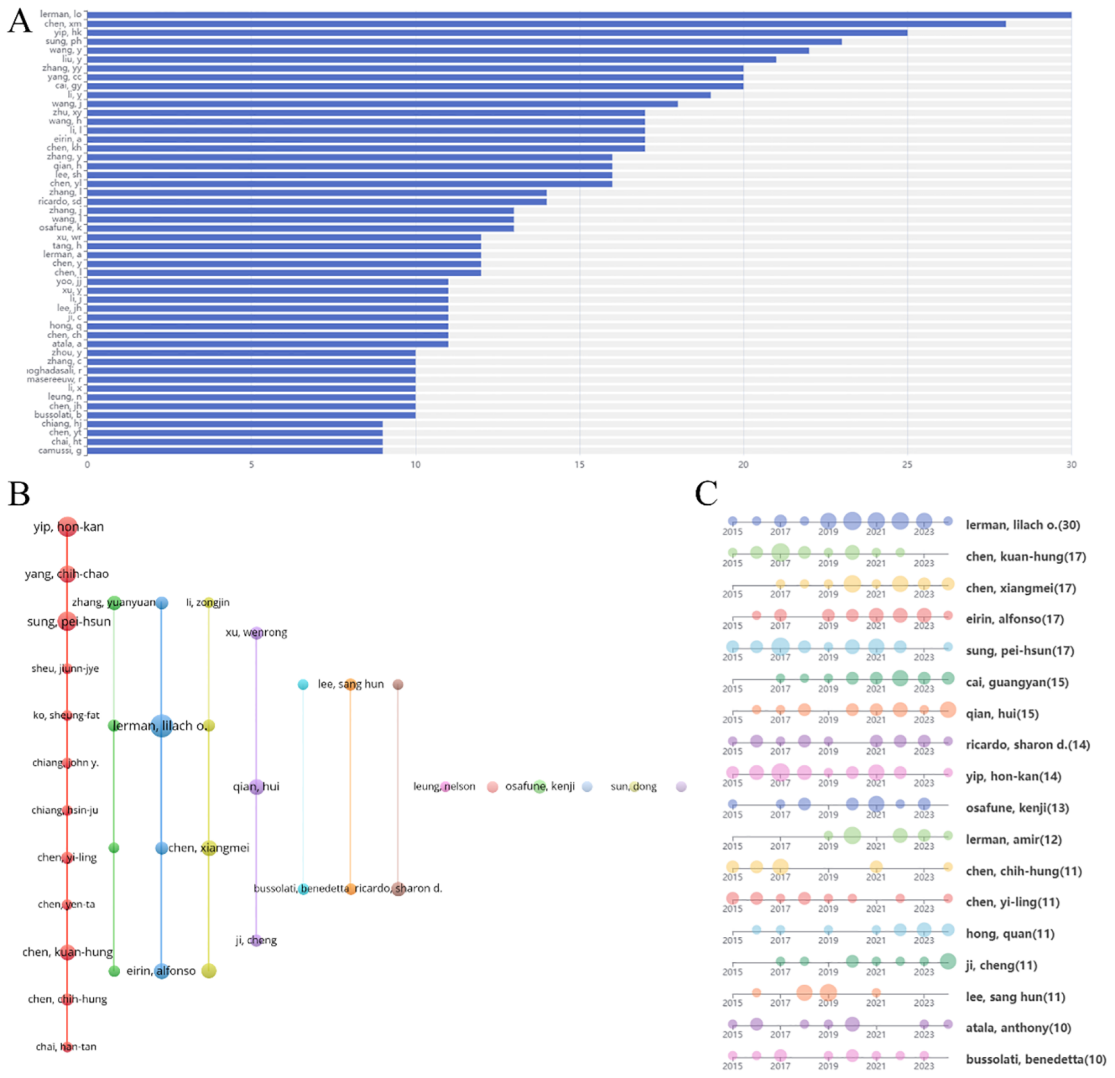
Analysis of authors engaged in research on stem cell therapy for kidney disease. **(A)** The top 50 authors’ publication volume chart. **(B)** Network map of authors’ collaboration. **(C)** Authors’ publication timeline.

A total of 9,741 authors were identified in this study. **Figure 6A** displays the top fifty authors by publication count, with Lerman LO leading with 30 publications, followed by Chen XM (28), Yip HK (25), Sung PH (23), and Wang Y (22). **Table 1** specifically lists the top 10 authors by number of publications and their most popular literature. The author collaboration network, shown in **Figure 6B**, reveals several prominent clusters of authors collaborating in stem cell therapy research for kidney disease. For example, Yip HK, Yang CC, and Sung PH form a significant collaboration cluster. Smaller clusters with fewer connections suggest weaker collaboration. Notably, there appears to be limited interaction between these author clusters. **Figure 6C** illustrates the annual publication trends of the authors over the past decade. For instance, Lerman LO has consistently published each year, Lee SH has not published in the past two years, and Lerman A began publishing in this field only in 2019.

**Table 1:** The top 10 authors by publication count and their most cited literature.

Rank	Author	Publications	Most cited publication	Year	Citations
1	Lerman LO	30	Cellular senescence: the good, the bad and the unknown (18)	2022	390
2	Chen XM	28	Supramolecular nanofibers containing arginine-glycine-aspartate (RGD) peptides boost therapeutic efficacy of extracellular vesicles in kidney repair (19)	2020	140
3	Yip HK	25	Combination of adipose-derived mesenchymal stem cells (ADMSC) and ADMSC-derived exosomes for protecting kidney from acute ischemia-reperfusion injury (20)	2016	189
4	Sung PH	23	Combined melatonin and exendin-4 therapy preserves renal ultrastructural integrity after ischemia–reperfusion injury in the Male rat (21)	2015	40
5	Wang Y	22	Exosomes secreted by human urine-derived stem cells could prevent kidney complications from type I diabetes in rats (22)	2016	215
6	Liu Y	21	Renoprotective approaches and strategies in acute kidney injury (23)	2016	89
7	Cai GY	20	IL-17A improves the efficacy of mesenchymal stem cells in ischemic-reperfusion renal injury by increasing treg percentages by the COX-2/PGE2 pathway (24)	2018	102
8	Yang CC	20	Combination of adipose-derived mesenchymal stem cells (ADMSC) and ADMSC-derived exosomes for protecting kidney from acute ischemia-reperfusion injury (20)	2016	189
9	Zhang YY	20	Beneficial effects of urine-derived stem cells on fibrosis and apoptosis of myocardial, glomerular and bladder cells (25)	2016	48
10	Li Y	19	Tranilast prevents renal interstitial fibrosis by blocking mast cell infiltration in a rat model of diabetic kidney disease (26)	2018	30

### 3.5 Analysis of cited literature and references

**Figure 7:**
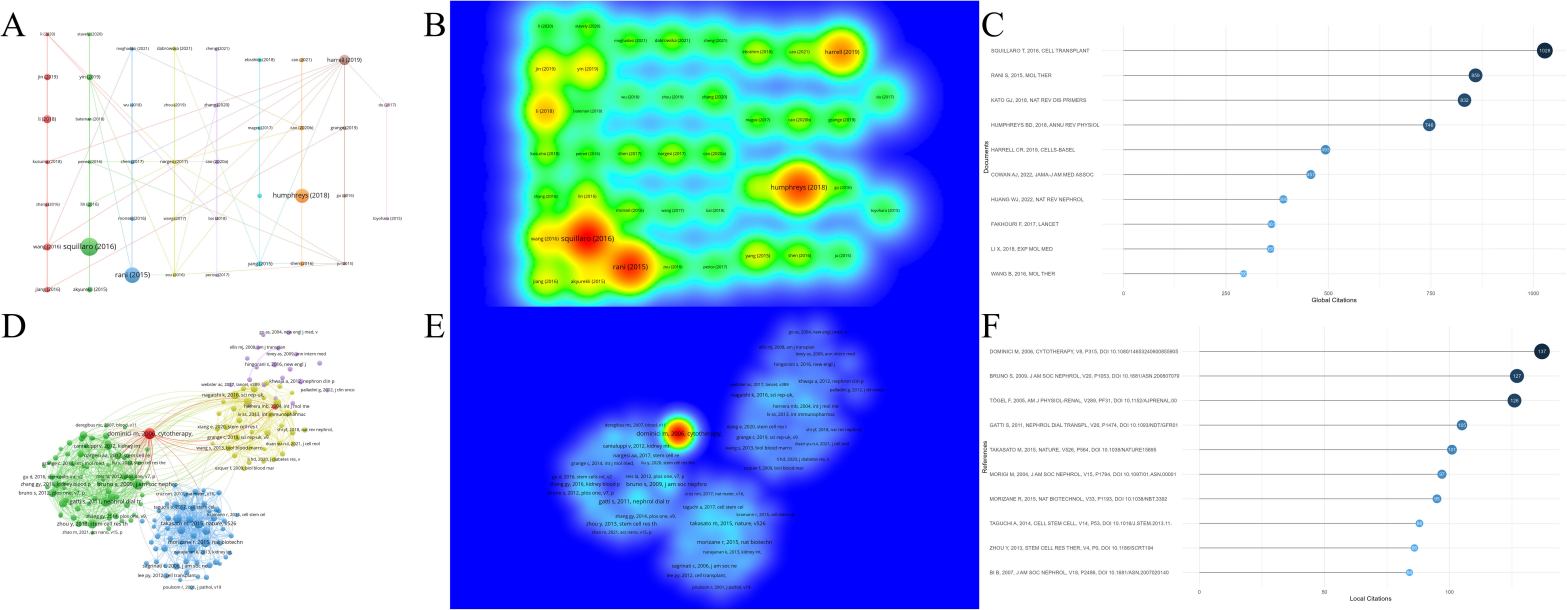
Analysis of cited literature and references in research on stem cell therapy for kidney disease. **(A)** Network map of cited literature. **(B)** Density visualization of cited literature. **(C)** The top 10 cited literature volume chart. **(D)** Network map of cited references. **(E)** Density visualization of cited references. **(F)** The top 10 cited references volume chart.

A higher number of citations generally indicates a more significant academic impact. Among the 1,874 documents included in this study, the article *“Clinical Trials with Mesenchymal Stem Cells: An Update”* (27), authored by Squillaro T et al. and published in *Cell Transplantation*, received the most citations and had the highest citation density (**Figures 7A–C**). This paper reviews the regenerative potential and clinical applications of MSCs, emphasizing their ability to differentiate into various cell types and their therapeutic uses in diseases such as graft-versus-host disease, diabetes, inflammatory conditions, and organ dysfunction. It also addresses challenges such as variability in MSC isolation and cultivation, critiques the heterogeneity of non-clonal cultures, and examines over 493 MSC-based clinical trials, highlighting both their therapeutic promise and ongoing challenges. The information of the top 10 most popular literature in this research field is listed in **Table 2**.

**Table 2:** The top 10 most cited literature in research on stem cell therapy for kidney disease.

Rank	Title	First Author	Journal	Citations	Year
1	Clinical Trials with Mesenchymal Stem Cells: An Update (27)	Squillaro T	Cell Transplant	1028	2016
2	Mesenchymal stem cell-derived extracellular vesicles: toward cell-free therapeutic applications (28)	Rani S	Mol Ther	859	2015
3	Sickle cell disease (29)	Kato GJ	Nat Rev Dis Primer	832	2018
4	Mechanisms of renal fibrosis (30)	Humphreys BD	Annu Rev Physioll	746	2018
5	Mesenchymal stem cell-derived exosomes and other extracellular vesicles as new remedies in the therapy of inflammatory diseases (31)	Harrell CR	Cells	493	2019
6	Diagnosis and management of multiple myeloma: a review (32)	Cowan AJ	JAMA	457	2022
7	Cellular senescence: the good, the bad and the unknown (18)	Huang W	Nat Rev Nephrol	390	2022
8	Hemolytic uremic syndrome (33)	Fakhouri F	Lancet	361	2017
9	Exosomes from adipose-derived stem cells overexpressing Nrf2 accelerate cutaneous wound healing by promoting vascularization in a diabetic foot ulcer rat model (34)	Li X	Exp Mol Med	359	2018
10	Mesenchymal stem cells deliver exogenous MicroRNA-let7c via exosomes to attenuate renal fibrosis (35)	Wang B	Mol Ther	293	2016

Among all references in the 1,874 documents (**Figures 7D–F**), the article *“Minimal criteria for defining multipotent mesenchymal stromal cells: The International Society for Cellular Therapy position statement”* (36), authored by Dominici M et al. and published in *Cytotherapy* in 2006, stands out. This paper outlines standardized criteria for defining human MSCs, aiming to improve consistency across studies, facilitate MSC characterization, and promote data sharing among researchers. **Table 3** lists the information of the top 10 most cited references in this research field.

**Table 3:** The top 10 most cited references in research on stem cell therapy for kidney disease.

Rank	Title	First Author	Journal	Citations	Year
1	Minimal criteria for defining multipotent mesenchymal stromal cells. The international society for cellular therapy position statement (36)	Dominici M	Cytotherapy	137	2006
2	Mesenchymal stem cell-derived microvesicles protect against acute tubular injury (37)	Bruno S	J Am Soc Nephrol	127	2009
3	Administered mesenchymal stem cells protect against ischemic acute renal failure through differentiation-independent mechanisms (38)	Tögel F	Am J Physiol Renal Physiol	126	2005
4	Microvesicles derived from human adult mesenchymal stem cells protect against ischemia–reperfusion-induced acute and chronic kidney injury (39)	Gatti S	Nephrol Dial Transplant	105	2011
5	Kidney organoids from human iPS cells contain multiple lineages and model human nephrogenesis (40)	Takasato M	Nature	101	2015
6	Mesenchymal stem cells are renotropic, helping to repair the kidney and improve function in acute renal failure (41)	Morigi M	J Am Soc Nephrol	97	2004
7	Nephron organoids derived from human pluripotent stem cells model kidney development and injury (42)	Morizane R	Nat Biotechnol	95	2015
8	Redefining the *In vivo* origin of metanephric nephron progenitors enables generation of complex kidney structures from pluripotent stem cells (43)	Taguchi A	Cell Stem Cell	88	2014
9	Exosomes released by human umbilical cord mesenchymal stem cells protect against cisplatin-induced renal oxidative stress and apoptosis *in vivo* and *in vitro* (44)	Zhou Y	Stem Cell Res Ther	86	2013
10	Stromal cells protect against acute tubular injury via an endocrine effect (45)	Bi B	J Am Soc Nephrol	84	2007

**Figure 8:**
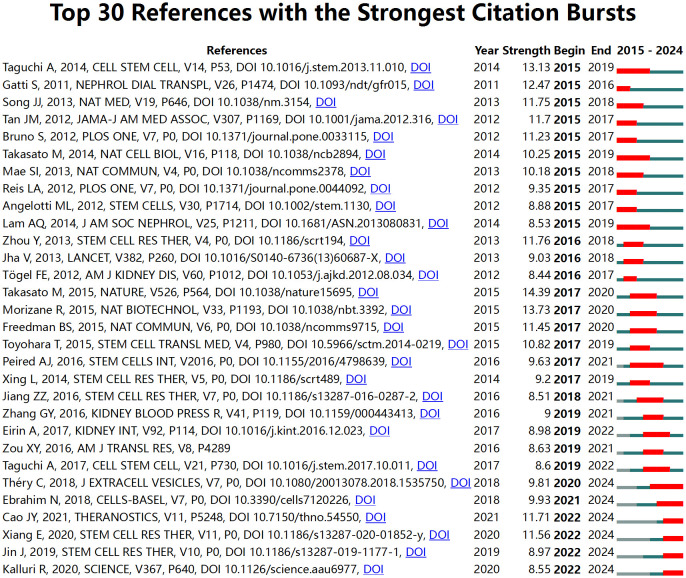
Top 30 references with the strongest citation bursts in research on stem cell therapy for kidney disease.

References with citation bursts indicate those highly cited over a specific period by researchers in the field. CiteSpace identifies the top thirty references in this area that experienced significant citation bursts (**Figure 8**). The red bars in **Figure 8** represent periods of notable citation bursts, with each bar corresponding to a specific year (46). Citation bursts were observed as early as 2011 and as recently as 2021. One reference, *“Redefining the in vivo origin of metanephric nephron progenitors enables generation of complex kidney structures from pluripotent stem cells”* (43) by Taguchi A et al., exhibited the strongest citation burst (strength = 13.13) during the period from 2015 to 2019. This paper explores the identification of the *in vivo* origin of metanephric nephron progenitors and demonstrates that these progenitors can be generated from pluripotent stem cells, leading to the creation of complex kidney structures *in vitro*. This finding provides new insights into kidney development and regenerative medicine.

### 3.6 Analysis of keywords

**Figure 9:**
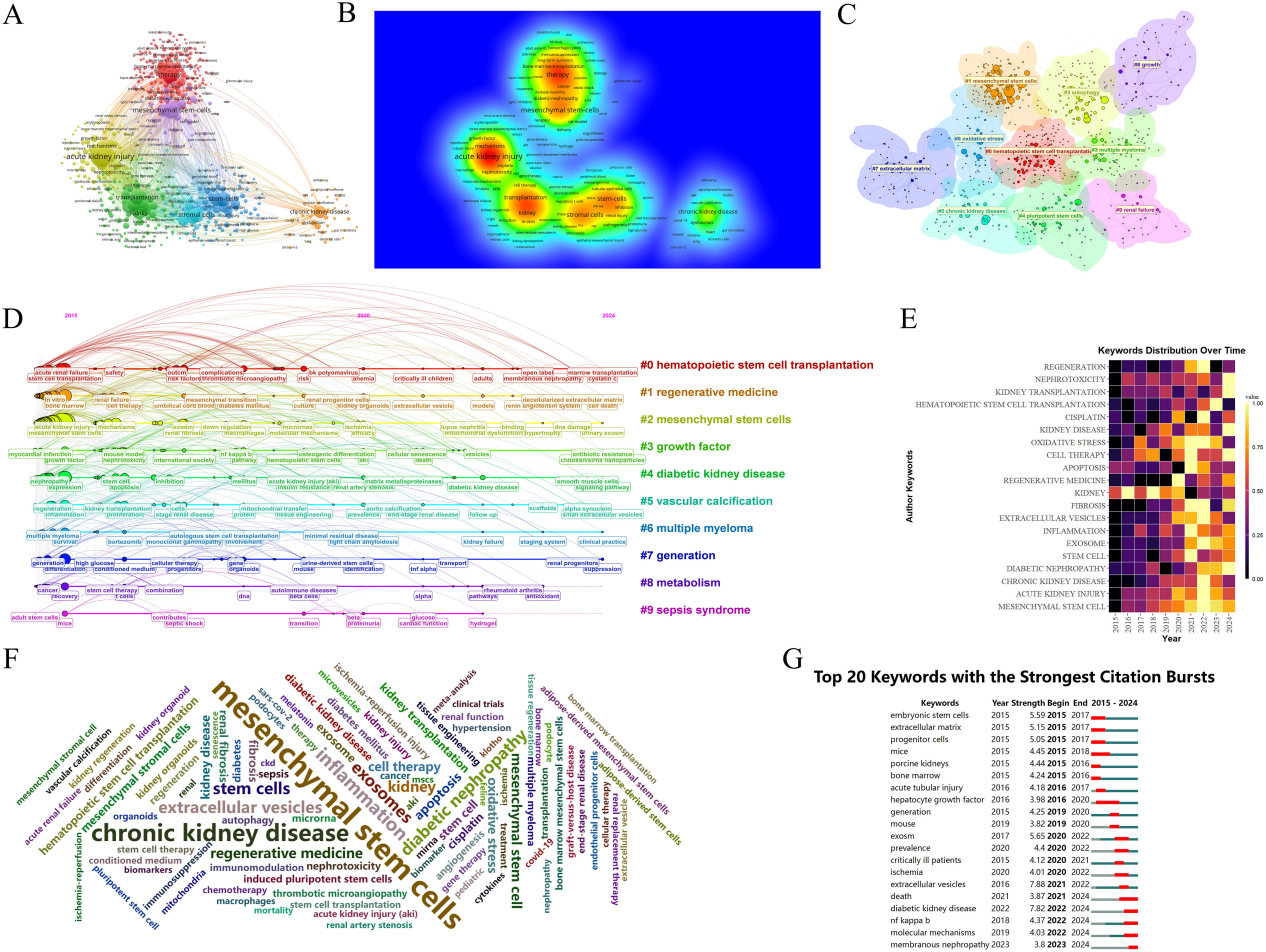
Analysis of keywords in research on stem cell therapy for kidney disease. **(A)** Occurrence of keywords. **(B)** Density visualization of keywords. **(C)** Keywords cluster diagram. **(D)** The timeline of keywords. **(E)** The annual heatmap of keywords. **(F)** The keywords cloud diagram. **(G)** Top 20 keywords with the strongest citation bursts in research on stem cell therapy for kidney disease.

Keywords are central to an article, and their visual analysis is crucial for identifying emerging trends and future directions in a research field. **Figures 9A, B** present the co-occurrence map of keywords, highlighting frequently mentioned terms such as MSCs, AKI, transplantation, stromal cells, and CKD. **Figure 9C** shows the formation of ten distinct clusters identified through a k-means clustering algorithm, covering topics such as hematopoietic stem cell transplantation, pluripotent stem cells, and the extracellular matrix.

The timeline analysis of keywords provides insight into the development and evolution of this research area. Using CiteSpace software, a timeline map was created (**Figure 9D**) to show the progression of keywords over time. The size of each node represents the frequency of keyword occurrence, while the position reflects the year, and the connecting lines highlight the co-occurrence relationships between keywords. CiteSpace categorizes the evolution of keywords into ten distinct timelines, which demonstrate a deepening focus on stem cell therapy for kidney disease in recent years. Recent research topics include marrow transplantation, decellularized extracellular matrix, smooth muscle cells, small extracellular vesicles, and more.

The annual heatmap of keywords (**Figure 9E**) provides an overview of the changing popularity of key terms. It shows that topics such as MSCs, apoptosis, and oxidative stress were prominent in earlier years, with a focus on the mechanisms of stem cells. More recent research has shifted toward diabetic nephropathy (DN) and kidney transplantation, suggesting an increased interest in applying stem cell therapy to specific kidney disease models or clinical patients.

**Figure 10:**
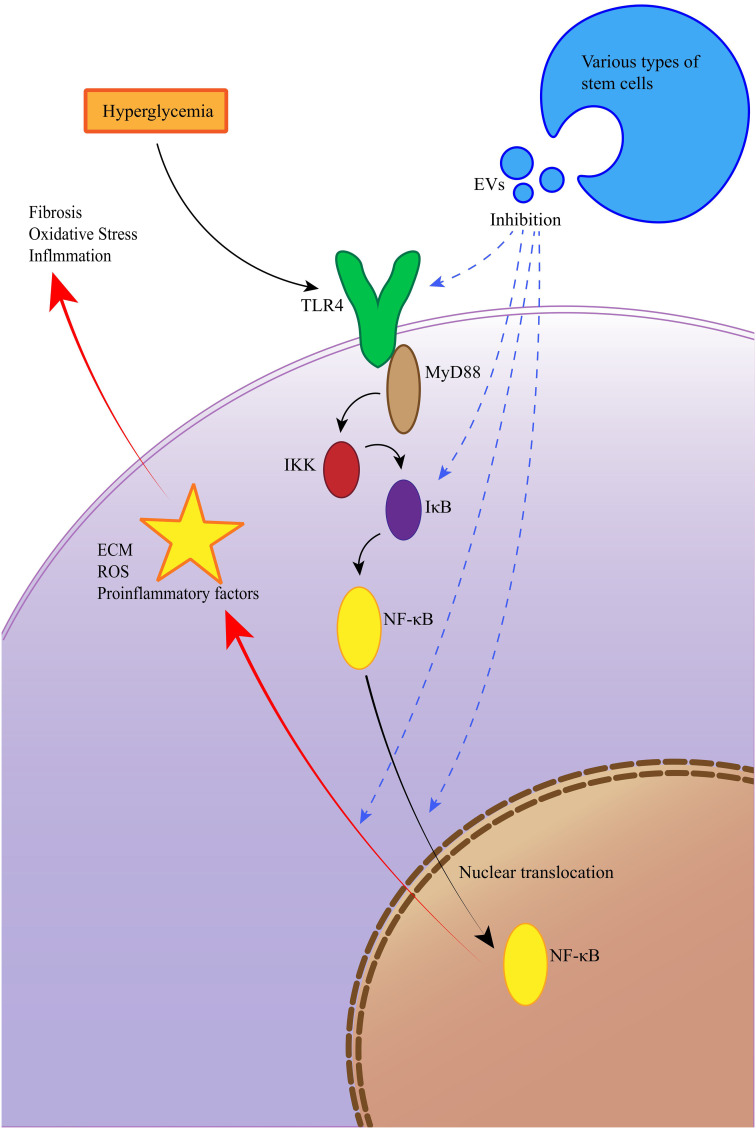
EVs ameliorate diabetic kidney disease via inhibition of the TLR4/NF-κB signaling pathway.

In the keyword cloud diagram (**Figure 9F**), the size of each keyword reflects its frequency of occurrence. This visualization indicates that MSCs and CKD remain dominant research areas within the field. The keyword burst chart (**Figure 9G**) highlights extracellular vesicles as the term with the strongest citation burst (strength = 7.88), showing high intensity from 2021 to 2022. The second highest burst is for diabetic kidney disease (DKD) (strength = 7.82), expected to peak from 2022 to 2024. This aligns with the trends shown in the annual heatmap (**Figure 9E**). Moving forward, research may increasingly focus on DKD and membranous nephropathy (MN), potentially emphasizing the NF-κB (Nuclear Factor kappa-light-chain-enhancer of activated B cells) pathway. In recent years, the therapeutic potential of EVs therapy targeting the TLR4/NF-κB signaling axis in DKD. A hyperglycemic microenvironment triggers activation of TLR4 receptors and MYD88 adaptor proteins, thereby promoting NF-κB phosphorylation and nuclear translocation (47, 48). This cascade elicits release of proinflammatory cytokines and extracellular matrix (ECM) deposition (49), culminating in inflammatory responses, oxidative stress, and renal fibrosis that accelerate DKD progression (47). EVs mitigate DKD by inhibiting the TLR4/NF-κB pathway through cargo-mediated mechanisms (e.g., miRNA delivery), with key regulatory effects including suppression of TLR4/MYD88 phosphorylation, prevention of IκBα degradation and reduction in NF-κB nuclear translocation (50), blockade of NF-κB-driven TGF-β signaling and collagen synthesis (51), among others (**Figure 10**). More innovative and comprehensive research is still needed.

## 4 Discussion

### 4.1 General information

This study employed bibliometric methods to analyze the development and application of stem cell therapy for kidney disease over the past decade. Stem cell therapy for kidney diseases has garnered significant attention as a potential treatment for renal injury and chronic conditions. The origins of these therapies lie within the broader field of regenerative medicine, which aims to repair or replace damaged tissues using stem cells. The initial clinical applications of stem cells in kidney disease emerged from an increased understanding of the regenerative potential of both adult and embryonic stem cells. Early research focused on the plasticity of adult stem cells, suggesting that cells derived from sources such as bone marrow or adipose tissue could help repair kidney damage. These cells have the ability to regenerate kidney tubules, glomeruli, and even address microvascular damage (52).

We analyzed 1,874 articles on stem cell therapy for kidney disease published in the Web of Science Core Collection (WOSCC) from January 1, 2015, to December 31, 2024. Using bibliometric and visualization tools, including CiteSpace, VOSviewer, R-Bibliometrix, and Ldgas, we examined the research trends and advancements in the field. Our quantitative analysis provided basic information on publications, countries, authors, institutions, and journals. The results reveal a rapid growth in literature over the past decade, indicating that stem cell therapy for kidney disease are a prominent research topic, attracting increasing scholarly attention. Through statistical analysis of publication output by country and institution, we identified key contributors and analyzed their collaborative relationships.

China and the United States are the leading countries in stem cell research for kidney disease, likely due to the high prevalence of kidney disease in these regions and their robust research infrastructure. Among the top ten institutions by publication volume, three are from mainland China, three from Taiwan, three from the United States, and one from Australia. While these countries show significant collaboration, there is limited interaction between institutions from China and the United States, which could hinder the long-term development of academic research in this area. The reasons for the lack of collaboration between China and the United States in this field may include the following: firstly, from a broader perspective, Sino-U.S. political relations and diplomatic exchanges have experienced turbulence over the past decade, coupled with significant geographical distance, which have created tangible barriers to cross-border research exchanges as policy discrepancies and geographical separation hinder collaborative efforts between researchers and institutions; secondly, in terms of stem cell research, the United States maintains a relatively lenient regulatory environment designed to accelerate clinical translation of stem cell therapies, with the FDA employing a risk-based regulatory approach for cell therapy approvals that balances innovation with safety, thus facilitating the expansion of clinical trials for stem cell therapies in kidney disease (53), while China has introduced supportive policies since 2017 to promote industrialization of stem cell-based therapies but its regulatory system remains in a state of refinement (54), with policies prioritizing expanded treatment accessibility and standardized management in contrast to U.S. policies that emphasize technological innovation and international competitiveness, leading to divergent research priorities that diminish collaborative opportunities; additionally, the two nations operate under distinct ethical review mechanisms and legal frameworks for stem cell research, which may give rise to regulatory conflicts and hinder cross-border scientific exchanges. To foster collaboration, it is imperative to establish mutually recognized ethical review frameworks, harmonize data standards, and mitigate competition and conflicts by identifying complementary research agendas.

As highlighted in **Figure 4A**, Dr. Lerman LO has made substantial contributions to the field of stem cell therapy for kidney disease, particularly in studies exploring the potential and challenges of MSCs in the treatment of chronic renal failure (CRF) (55), ischemic kidney disease (56), and targeted therapeutic strategies (57–60). SQUILLARO T’s article (27), which has the highest citation count among the 1,874 included documents, reviews the regenerative potential and clinical applications of MSCs. In fact, much of the most-cited literature and references in this field are related to mesenchymal stem cells, underscoring their central role in kidney disease research. **Figure 5D** illustrates that research published in journals related to Molecular Biology, Genetics, and Health/Nursing/Medicine is frequently cited in medical and biological literature, reflecting the progress made in both clinical and scientific aspects of stem cell therapy for kidney disease.

### 4.2 Hotspots and frontiers

Keywords are crucial in identifying the core areas of research, and their analysis can reveal emerging trends in a field. MSCs have emerged as a central focus of research in stem cell therapy for renal diseases, showcasing promising outcomes in preclinical and clinical investigations, notably in the management of renal fibrosis, tubular injury, and inflammatory responses. The renal therapeutic efficacy of MSCs is not mediated by direct differentiation into renal parenchymal cells, but primarily through paracrine secretion of bioactive molecules, which modulates renal fibrogenesis, inflammatory cascades, and immune homeostasis (61). This paracrine-mediated mechanism underscores the central tenet of their therapeutic action. For example, single-cell RNA sequencing has demonstrated that MSCs therapy significantly modulates the transcriptional profile of renal tubular epithelial cells (TECs) in AKI (62), inhibiting cellular apoptosis and promoting proliferation, which is advantageous for the treatment of renal ischemia-reperfusion injury. During the progression from AKI to CKD, MSCs suppress the transdifferentiation of pericytes into myofibroblasts, reducing excessive extracellular matrix deposition and thereby decelerating the progression of renal fibrosis following AKI (63). In terms of anti-inflammatory mechanisms, MSCs exert their effects by upregulating anti-inflammatory cytokines (e.g., IL-10), regulating T-cell responses (64), and inducing macrophage polarization from the M1 to M2 phenotype (65), among other pathways. These collective actions facilitate the restoration of renal immune homeostasis and contribute to the amelioration of renal injury. As the most studied stem cell type in this field, MSCs have numerous research applications. For instance, a recent study examined the combined effect of MSCs and all-trans retinoic acid (ATRA) in treating AKI. The results showed that ATRA pre-treatment improved MSC survival and homing abilities, enhancing therapeutic efficacy. This combination therapy resulted in better renal function recovery, reduced inflammation, and promoted tissue repair compared to MSCs alone (66).

In addition to MSCs, a range of other stem cells are being actively explored for kidney therapies. For instance, induced pluripotent stem cells (iPSCs) have demonstrated notable potential in the repair of renal injury. iPSCs can be directed to differentiate into renal progenitor cells—a cell type rarely found in renal tissue. These progenitors undergo ex vivo expansion and subsequent differentiation into functional renal lineages, thereby generating complex three-dimensional kidney organoids containing nephron-like architectures (67, 68). These organoids encompass key renal cell lineages—including podocytes, renal tubular epithelial cells, and renal interstitial cells—and even exhibit features of early/neonatal vascularization (69, 70). *In vitro*, these constructs recapitulate select renal functions, such as displaying filtration capacity (68). Compared with traditional two-dimensional cell cultures, iPSC-derived kidney organoids more accurately replicate the structural and functional attributes of native kidneys (71). As such, these organoids have emerged as a novel and robust tool for investigating diverse renal pathologies. What makes iPSCs particularly exciting is their potential for personalized medicine, as patient-specific iPSCs can be created, offering tailored treatments for various kidney diseases (72). Patient-specific iPSCs can be generated from the patient’s somatic cells (73, 74), which inherently harbor the patient’s genetic background, including disease-associated mutations. By differentiating these iPSCs into renal cell lineages (e.g., renal tubular cells) or generating kidney organoids, this approach enables the accurate recapitulation of the patient’s disease phenotype *in vitro* (75, 76). This ‘disease-in-a-dish’ model affords an unprecedented platform for the systematic investigation of pathogenesis in individual-specific renal diseases, serving as the cornerstone for elucidating interindividual disease heterogeneity. These iPSC-derived renal cells or organoids can be directly utilized for drug response profiling, thereby exhibiting substantial potential in personalized drug screening and drug toxicity assessment (77). This approach enables the systematic evaluation of efficacy and safety for diverse treatment regimens in *in vitro* patient-specific models—including pharmacodynamic and toxicological analyses—thereby providing clinicians with personalized therapeutic guidance to mitigate the risk of administering ineffective or harmful interventions. Notwithstanding their promise, several critical challenges must be addressed before iPSCs can be translated into clinical applications: existing kidney organoids fail to recapitulate the complete developmental integration of renal architectures, harbor restricted cellular heterogeneity, and exhibit incomplete functional maturation (70); current models cannot generate perfusable vascular networks, thereby impeding organoid growth, restricting size expansion, and compromising filtration capacity—despite exhibiting filtration activity, the filtrate lacks a defined excretory pathway (69, 78). These limitations severely restrict their capacity for *in vivo* integration and functional restoration. Strategies for engineered vascularization to optimize organoid-guided differentiation, self-organization, maturation, and vascularization cascades represent a pivotal research avenue to augment the therapeutic utility of organoids (79). Embryonic stem cells (ESCs) are another powerful source of kidney cell types, preclinical models suggest ESCs could play a role in kidney regeneration, potentially becoming part of future therapies for kidney diseases (10). ESCs can also differentiate into renal progenitor cells for damaged cell replacement or renal organoid generation. Beyond their direct differentiation and reparative functions, ESCs or their derived renal progenitors may secrete paracrine factors that activate endogenous repair mechanisms upon transplantation. This involves stimulating the proliferation and differentiation of resident stem/progenitor populations (e.g., Sox9-positive cells) in the kidney, thereby mobilizing the organ’s intrinsic repair capacity against injury (80). However, ESCs are derived from early human embryos (inner cell mass of blastocysts), a process requiring embryo destruction that ignites ethical controversies regarding embryonic moral status. Due to these ethical constraints, many nations and regions impose strict restrictions on ESC research funding and clinical translation, significantly impeding its advancement (81). In contrast, iPSCs—derived from somatic cells—circumvent ethical dilemmas while exhibiting ESC-comparable multipotent differentiation potential, rendering them preferable to ESCs in renal disease research. Hematopoietic stem cells (HSCs) exhibit a unique duality in the context of kidney disease. On one hand, hematopoietic stem cell transplantation (HSCT) serves as an effective and even curative modality for hematological malignancies, genetic blood disorders, primary immunodeficiencies, and selected non-malignant hematopathies (82). However, HSCT recipients face a significant risk of renal injury, with these complications associated with substantial mortality and a high likelihood of progression to ESRD (83). This underscores the imperative for strategies to mitigate renal injury during and after HSCT, establishing this as a critical research frontier (84, 85). On the other hand, HSCs themselves possess inherent potential for renal repair. Human kidneys may harbor HSCs with hematopoietic competency (86). Under defined conditions (e.g., post-transplantation), kidney graft-derived HSCs can achieve long-term engraftment in the recipient bone marrow, establishing high-level multilineage donor hematopoietic chimerism—thereby potentially promoting immune tolerance to the transplanted organ. This mechanism involves “bone marrow niche remodeling” and the facilitatory effect of donor graft-versus-host disease (GVHD)-reactive T cells on donor HSC implantation (86). These findings suggest that HSCs—particularly kidney-derived populations—play a unique role in tolerance induction and potential modulation of the renal microenvironment. Elucidating this dual nature is pivotal for balancing therapeutic efficacy and risks, as well as for developing safer and more effective interventions for kidney disease. Autologous renal stem cells refer to stem cells or progenitor cells isolated from the patient’s own kidneys, exhibiting the potential for self-renewal, proliferation, and multidirectional differentiation to generate various renal cell types (e.g., renal tubular epithelial cells), thereby contributing to renal tissue repair and regeneration. These cells include specific populations identified in adult kidney tissue, such as Sca1^+^Oct4^+^-marked renal stem/progenitor cells (87) and human CD133^+^CD24^+^ renal progenitor cells (88), which have shown promise as autologous sources in tissue engineering. Notably, cells derived from diseased kidneys (e.g., in chronic kidney disease or end-stage renal disease) can also serve as autologous therapeutic sources, leveraging their inherent advantage of avoiding immune rejection (89). Compared with other stem cell therapies, autologous renal stem cells minimize immune rejection risks and enable the construction of kidney organoids (90) or advanced applications in tissue engineering and regenerative medicine. For instance, inducing autologous renal stem cells to differentiate into renal progenitor cells and combining them with scaffold materials facilitate the development of complex bioengineered renal structures (90). Overall, research into the types of stem cells and the mechanisms behind their effectiveness in treating kidney diseases is ongoing.

Traditional stem cell therapy typically involves the direct transplantation of live stem cells, believed to repair kidney tissue by differentiating into various kidney cell types, secreting trophic factors, and modulating the immune response. MSCs are the most commonly used in these therapies. However, cell-based therapy entails substantial risks: transplanted viable cells may elicit host immune responses, necessitating long-term administration of immunosuppressive agents (91); viable cells pose potential risks of aberrant proliferation or malignant transformation (92); and procedures for cell preservation, transportation, and implantation involve significant technical challenges and costs (93). In contrast, extracellular vesicle (EV) therapy focuses on leveraging vesicles secreted by stem cells, which carry bioactive cargoes—including proteins, nucleic acids (RNA), and lipids—that mediate analogous regenerative effects without requiring whole-cell transplantation. Lacking intact cellular structures, EVs exhibit low immunogenicity, obviate the need for long-term immunosuppression (92), and ensure enhanced safety profiles. EVs from various sources, including MSCs, endothelial cells, and kidney cells, have been shown to reduce inflammation, promote tissue repair, and regulate fibrosis (94, 95). A particular area of interest within EVs therapy is exosomes, small extracellular vesicles that carry bioactive molecules which can influence recipient cells’ functions. Exosome-based therapies offer several advantages, including avoiding direct stem cell transplantation, reducing immune rejection, and enabling targeted delivery of bioactive molecules to kidney cells (96), making it a promising alternative to traditional stem cell therapies. Several stem cell-based therapies utilizing EVs are under investigation, such as MSC-derived small extracellular vesicles (sEVs), which have demonstrated beneficial effects in treating kidney diseases, including both AKI and CKD. These vesicles help modulate inflammation, reduce fibrosis, and enhance tissue regeneration in the kidney (97–99). Urinary extracellular vesicles (uEVs) are also being explored as a promising source of diagnostic and therapeutic material for kidney diseases. uEVs derived from renal tubular cells and other renal parenchymal cells exhibit molecular profiles that non-invasively mirror the severity of renal injury (100). Moreover, as diagnostic biomarkers, uEVs demonstrate superior performance compared to conventional serum creatinine assays (91). Endothelial cells, which line blood vessels, have also been identified as a source of therapeutic EVs for kidney diseases. These EVs help regulate renal blood flow, reduce inflammation, and promote endothelial repair, all of which are essential for kidney health (101). Endothelial progenitor cell-derived extracellular vesicles (EPC-EVs) emerge as a promising candidate for cell-free therapeutic strategies, particularly in the management of renal ischemia-reperfusion injury (IRI). IRI represents a leading cause of acute kidney injury (AKI), yet effective therapeutic strategies for this condition remain limited. EPC-EVs hold significant promise for renal IRI management, primarily due to their ability to orchestrate angiogenic and regenerative signaling cascades. This therapeutic approach capitalizes on the intercellular communicative functions of EVs to mitigate endothelial damage and promote renal reparative responses (102). Additionally, extracellular vesicles derived from renal epithelial cells have shown the ability to promote regeneration in damaged kidney tissue, carrying protective factors that enhance the survival and proliferation of kidney cells, particularly in AKI. EVs derived from renal tubular cells have demonstrated the potential to repair damaged renal tubules and improve kidney function, representing a novel therapeutic strategy for AKI and CKD (103). In AKI, renal tubular epithelial cell-derived EVs mitigate cellular apoptosis and mitochondrial damage by activating the autophagic signaling pathway (104). Platelet-derived EVs have also attracted attention for their role in kidney injury repair, as they are rich in growth factors and cytokines that stimulate tissue repair and regeneration. Studies have indicated that platelet-derived EVs can enhance endothelial repair and reduce fibrosis in kidney tissue (93, 105). While stem cell-based therapies are subject to stringent regulatory approval processes due to the risks associated with live cell use, EV therapy, being acellular, may face fewer regulatory hurdles, though it still requires careful assessment for safety and efficacy. The relatively lower regulatory burden could accelerate EVs-based therapies for kidney disease development and approval. However, further clinical trials and standardization of EVs isolation and delivery methods are necessary to fully realize the potential of EVs in kidney disease therapy.

Stem cell therapy has become a focal point in the treatment of various kidney diseases due to its potential for tissue repair and functional restoration. AKI, a condition commonly caused by ischemia, toxins, or infections, is a primary target for these therapies. Researchers have focused on how stem cells can aid renal tissue repair and improve kidney function in AKI. A study compared different stem cell types found that MSCs—particularly those derived from bone marrow or adipose tissue—significantly enhanced kidney function and promoted tissue regeneration in AKI models (106). Recent investigations have revealed that MSCs suppress pyroptosis by modulating mitochondrial function in renal tubular epithelial cells, thereby ameliorating AKI and impeding the progression to long-term renal fibrosis (107). This mechanism not only facilitates acute injury repair but also confers protective effects against CKD. EVs derived from induced mesenchymal stem cells (iMSCs) have been demonstrated to impede the transition of AKI to CKD, marking an innovative breakthrough in cell-free therapeutic paradigms (108). These findings suggest that MSCs may offer superior potential for acute renal recovery. The application of stem cell therapy in the context of CKD is classified into several distinct categories. DKD and MN have emerged as heat areas of study. MSCs may ameliorate DKD by modulating renal fatty acid oxidation. Dyslipidemia represents a cardinal feature of DKD, and the therapeutic action of MSCs may be intricately linked to the regulation of these metabolic derangements (109, 110). Optimizing renal vascularization represents a key therapeutic objective in DKD, and MSCs infusion has emerged as a promising novel strategy for restoring injured vascular architecture and function (111). Given the multifactorial pathogenesis of DKD, another research frontier focuses on exploring combinatorial therapies stem cells with other pharmacotherapies. A compelling example is the MSCs + empagliflozin combination, which has demonstrated synergistic renal protective effects exceeding those of monotherapies in preclinical animal models (112). Notwithstanding the current scarcity of clinical evidence, MSCs have exhibited promise in enhancing renal function within preclinical animal models of DKD (113). The current focus of stem cell therapy in MN primarily centers on mechanistic investigations and therapeutic explorations of secondary MN following allogeneic HSCT, rather than direct stem cell therapy as a standalone modality. The risk of MN in patients following allogeneic HSCT is significantly increased, representing the most common *de novo* glomerular disease after transplantation (114). For MN following allogeneic HSCT, no standardized treatment protocol exists. Traditional immunosuppressive therapies exhibit limited efficacy and may exacerbate the risk of infection (115). Future investigations may delve into the role of stem cells in mediating renal tissue repair. Other conditions, such as kidney fibrosis (116) and focal segmental glomerulosclerosis (FSGS) (117), are also being investigated in relation to stem cell therapy. Research on stem cell therapy for kidney transplantation is also progressing, emphasizing regenerative therapies to improve graft survival and kidney function (118).

Despite the advancements in stem cell therapy for kidney diseases, translating these therapies into clinical practice still poses significant challenges. Presently, poor stem cell viability and suboptimal engraftment efficiency *in vivo* represent critical barriers to their clinical translation, particularly in the context of renal injury, where impaired cell survival substantially hampers therapeutic efficacy (119). Current research efforts are focused on developing novel delivery methodologies and cell survival strategies, including the optimization of cell sources, dosage regimens, and administration routes (e.g., leveraging biomaterials or genetic engineering approaches) to enhance stem cell viability and integration potential (120). In addition to the aforementioned challenges, stem cell transplantation also entails inherent risks, such as the fact that MSCs currently represent the most intensively researched type of stem cells in this field; however, MSCs exhibit a low enrichment rate in the kidneys when administered via intravenous injection (121), and even when delivered to the target treatment site through various methods, they are often washed away or undergo necrosis (122). Moreover, the pathological environment of kidney disease further impairs the survival and proliferation rates of transplanted MSCs, thereby limiting their regenerative potential. In patients with CKD, the viability and proliferative capacity of MSCs are reduced due to prolonged exposure to uremic toxins, chronic inflammation, and oxidative stress, thereby limiting their therapeutic efficacy (123, 124), while AKI, which shares a similar pathological environment, also hinders the application of MSCs (125). The traceability of MSCs remains a critical challenge. To address this, numerous studies employ cell labeling strategies, such as tagging MSCs with magnetic nanoparticles (e.g., cobalt ferrite NPs) for subsequent tracking via visualization techniques (e.g., magnetic resonance imaging, MRI) (126). However, tracking unlabeled MSCs in their native state following administration remains inherently difficult, limiting the ability to characterize their *in vivo* behavior without exogenous markers. In clinical applications, induced pluripotent stem cells (iPSCs) face notable tumorigenicity risks, as undifferentiated iPSCs or incompletely differentiated cells may give rise to teratomas or other neoplastic growths (127). Even when utilizing iPSC-derived podocytes or glomerular tissues, careful consideration must be given to the long-term stability risks following implantation (128). Additionally, ethical concerns associated with embryonic stem cells and other cell sources require urgent resolution and consensus-building to address the associated moral and regulatory challenges. Future research agendas will focus on: Elucidating the immune-modulatory mechanisms of stem cells and developing genetic engineering techniques to mitigate rejection risks; Conducting rigorous preclinical and clinical safety assessments; Establishing standardized ethical guidelines for stem cell source procurement and application. From a disease-oriented perspective, the multifaceted pathogenesis of kidney diseases—encompassing chronic inflammation and fibrosis—imposes significant constraints on the therapeutic efficacy of stem cells, as current therapeutic modalities fall short of completely reversing the disease progression. Current research endeavors are centered on unraveling the regenerative mechanisms of stem cells and developing integrative strategies that combine pharmacotherapies to address these complex pathologies (129). In the realm of therapeutic technology, the restricted capacity for real-time *in vivo* visualization of stem cells has posed a significant barrier to the evaluation of treatment mechanisms and safety, thereby impeding clinical translation. Current research efforts are concentrated on the development of advanced imaging and tracking technologies—such as molecular imaging or nanoparticle labeling—to facilitate superior monitoring of stem cell distribution, migration, and biological behavior, with the aim of optimizing treatment strategies (130). These concerted efforts are directed at translating the promise of stem cell therapy into tangible clinical applications. However, several technical bottlenecks—including those related to cell survival, immune rejection, and *in vivo* tracking—remain to be surmounted. In exosome research, investigations have been predominantly focused on preclinical studies and early-phase clinical trials, with translational challenges persisting in bridging experimental findings to clinical practice. Notwithstanding preclinical evidence in animal models demonstrating that exosomes exert anti-inflammatory and anti-fibrotic effects in AKI and CKD, clinical evidence in humans remains scarce (131). Currently, this field of research is lacking support from large-scale clinical trials, and existing studies have failed to clearly define dosage, administration frequency, and long-term safety parameters, thereby impeding the standardization of therapeutic regimens (132). Current research efforts are concentrated on addressing gaps in clinical evidence, establishing standardized manufacturing systems, and deepening mechanistic investigations. Breakthroughs in these domains will directly influence the clinical feasibility of exosome-based therapies for kidney diseases, as they underpin the transition from preclinical research to routine clinical application. However, it is crucial to emphasize that these limitations and challenges represent an inevitable phase in the evolution of a burgeoning field. As such, the feasibility of stem cell-based therapies for renal diseases should not be summarily dismissed.

Research on various types of stem cells has become increasingly comprehensive, with new therapeutic approaches being developed. More kidney disease models are being explored, expanding the scope of potential treatments. However, further in-depth investigation and clinical studies are essential to fully realize the clinical potential of these therapies.

### 4.3 Limitations

The research data were sourced from the WOSCC, with 1,874 publications included in the analysis. Other types of literature and non-English sources were excluded, which introduces a degree of source bias. The WOSCC, as a core authoritative database, facilitates retrieval of high-quality studies while filtering out low-quality literature that could compromise analytical validity. However, this exclusive approach may inadvertently omit relevant publications. Although multi-database analyses theoretically enhance comprehensiveness, interoperability challenges arising from heterogeneous data formats often introduce analytical noise without substantive value addition. Future investigations will integrate high-value platforms (e.g., PubMed and Scopus) to enable more comprehensive bibliometric analyses.

## 5 Conclusions

This study utilized bibliometric methods to assess the development and application of stem cell therapy for kidney disease over the past decade. Through statistical analysis and evaluation of information from different countries, institutions, authors, and journals, and using tools such as CiteSpace, VOSviewer, R-Bibliometrix, and Ldgas, a total of 1,874 articles were analyzed to track trends and advancements in the field. The findings reveal significant growth in research output, with China and the United States emerging as the leading contributors. Key institutions and influential authors were identified, though limited collaboration between Chinese and American institutions could hinder further progress. The primary focus of stem cell therapies remains on MSCs. Additionally, other stem cell types, including iPSCs, renal progenitor cells, and ESCs, are being actively explored for their regenerative potential. A growing area of interest is EVs, which offer a promising alternative to direct stem cell transplantation by carrying bioactive molecules that promote tissue repair and regeneration. DKD may become widespread diseases or models for future research. Despite the promising preclinical findings, challenges remain in translating these therapies to clinical practice, particularly regarding safety, efficacy, and regulatory approval. Based on the findings of the bibliometric analysis, we propose the following recommendations to advance the field: establish a more internationally unified ethical framework; develop standardized international stem cell databases—with particular emphasis on Sino-U.S. collaboration—to promote data sharing, mitigate competitive pressures, and foster collaboration; and conduct large-scale, multicenter randomized controlled trials in emerging research frontiers, notably DKD and EVs. Achieving these objectives will necessitate concerted efforts from researchers and clinicians, requiring interdisciplinary collaboration to bridge translational gaps.

## Data Availability

The datasets presented in this study can be found in online repositories. The names of the repository/repositories and accession number(s) can be found in the article/supplementary material.
